# Effect of Reinforced Oral Hygiene on Periodontally Healthy Dental Students: A Four-year Follow-up Clinical Trial

**DOI:** 10.3290/j.ohpd.a45434

**Published:** 2020-10-27

**Authors:** Marie-Caroline Baudrion, Céline Bories, Assem Soueidan, Bénédicte Enkel, Xavier Struillou, Zahi Badran

**Affiliations:** a Dental Surgeon, Department of Periodontology, UIC 11, CHU Nantes, Rmes Lab, Inserm U1229, Faculty of Dental Surgery, University of Nantes, France. Performed the experiments in partial fulfillment of requirements for a MSc, wrote the manuscript.; b Dental Surgeon, Department of Periodontology, UIC 11, CHU Nantes, Rmes Lab, Inserm U1229, Faculty of Dental Surgery, University of Nantes, France. Performed the experiments in partial fulfillment of requirements for a MSc.; c Professor, Department of Periodontology, UIC 11, CHU Nantes, Rmes Lab, Inserm U1229, Faculty of Dental Surgery, University of Nantes, France. Contributed to the hypothesis, experimental design, data interpretation, progress supervision, and reviewing the manuscript.; d Associate Professor, Department of Restorative Dentistry and Endodontics, Faculty of Dental Surgery, University of Nantes, France. Contributed to experimental design and statistical analysis.; e Associate Professor, Department of Periodontology, UIC 11, CHU Nantes, Rmes Lab, Inserm U1229, Faculty of Dental Surgery, University of Nantes, France. Associate Professor, contributed to data interpretation and progress supervision.; f Professor, Department of Periodontology, UIC 11, CHU Nantes, Rmes Lab, Inserm U1229, Faculty of Dental Surgery, University of Nantes, France; Faculty of Dentistry, McGill University, Montreal, Canada; College of Dental Medicine, University of Sharjah, Sharjah, UAE. Contributed to experimental design, data interpretation, progress supervision, data interpretation, and reviewing the manuscript.

**Keywords:** bleeding on probing, oral hygiene, plaque index, probing depth, sulcus

## Abstract

**Purpose:**

In periodontally healthy individuals, mean crevicular depth ranges from 1 to 3 mm. This depth threshold has been used as an indicator to differentiate a physiological dentoalveolar sulcus from a periodontal pocket needing further treatment. Because many studies have shown the important contribution of oral hygiene status to periodontal health, the purpose of this study was to explore the clinical effect of reinforced oral hygiene on the periodontal status of periodontitis-free dental students.

**Materials and Methods:**

In our longitudinal observational clinical study, we assessed the periodontal status of healthy individuals attending the dental school by measuring the periodontal pocket depth, bleeding on probing, and plaque index. The follow-up reassessment was carried out four years later at the end of the dental curriculum.

**Results:**

The study showed that oral hygiene improvement led to a slight but significant reduction in the mean sulcus depth (-0.049 mm; p<0.0001).

**Conclusions:**

Reinforcement of oral hygiene contributes to the reduction of probing depth even in periodontally healthy patients.

Periodontal diseases have been associated with several systemic diseases and conditions.^13^,^17^ Positive correlations between plaque, inflammation and attachment loss have been reported.^19^,^20^ Oral hygiene (OH) plays a major role in maintaining a healthy periodontium^2^,^15^ and supposedly in the prevention of associated systemic diseases.^14^,^16^ Hence, rigorous maintenance of good OH can help to prevent the onset and development of periodontal diseases.

Many studies have described the effect of reinforced OH on the success and efficacy of periodontal treatments.^1^,^3^ For instance, meticulous supragingival plaque control can significantly reduce probing depth in shallow pockets by quantitatively and qualitatively influencing subgingival microbiota and biomarkers of inflammation.^8^ However, few trials exist on the impact of increased OH on already healthy periodontium. 

Data from clinical studies suggest that psychological variables play a role in adherence to and compliance with optimal oral health behaviour.^10^ Motivational interviews, for example, are a promising approach for improving compliance with plaque control.^7^ Also, a greater knowledge and awareness of periodontal diseases and the different methods to enhance oral health leads to a better understanding of the pathology and may help the patient become more engaged in treatment. Therefore, these OH parameters could ultimately increase the success rates of periodontal treatments. These are variables and interventions that, as future practitioners, dental students should become familiar with during the course of their studies and should apply themselves on a daily basis to maintain a healthy periodontium. 

Measures such as periodontal pocket depth (PPD), bleeding on probing (BoP) and O’Leary Plaque Index (PI) are commonly taken to assess and monitor periodontal status.^14^Generally, a healthy periodontium is characterised at the midline by a 0.5-1 mm sulcus depth, increasing to 3 mm at proximal sites,^5^ no bleeding upon probing and a plaque index <20%. It has also been established that even in clinically healthy periodontium, an inflammatory infiltrate is histologically observed in the subepithelial lamina propria,^15^ suggesting that histological and clinical improvement is still possible at the periodontal level. 

The aim of this study was to investigate the changes in a clinically healthy periodontium in a population of young, healthy dental students after reinforced dental hygiene during four years of dental education.

## Study Population and Methods

The study was conducted at the Department of Periodontology of Nantes University Hospital from January 2009 to June 2013, in compliance with the local clinical investigation unit guidelines and acceptance (UIC 11, Odontology, CHU de Nantes).

## Study Population

Systemically healthy students from the undergraduate dental curriculum in 2009 at the University of Nantes, France, were considered eligible for inclusion in this study. All students were volunteers, as an announcement was made to the whole class year, and the experimental protocol was explained in detail before the study began. All participants expressed their informed consent to participate in the study, agreed to attend the follow-up visit four years later (during the sixth and last year of the curriculum), and had at least six teeth, including the first or second molars in each quadrant. In respect of these inclusion criteria, the final screening yielded a sample of 25 participants aged 19 to 23 at the time of the first examination session. 

## Study Protocol

All individuals were examined twice: the first visit (V1) was during their second year of the undergraduate program (January 2009–June 2009) and before the scheduled courses on OH instructions. The follow-up visit (V2) was carried out with the same student group during their last year in the dental curriculum (September 2012–June 2013), after adequate OH instructions and four years of acquiring considerable knowledge and awareness of periodontal diseases, their prophylaxis and treatment modalities. In this study, professional cleaning was not part of the protocol. The measurements at baseline were recorded by an experienced examiner (postgraduate dental resident) trained in the use of the electronic probe. At the follow-up visits, a different yet equally trained examiner took the measurements. The latter was blinded for the baseline periodontal examination charts. Before each visit, students were informed not to modify their usual OH habits.

At each visit, the students were given a questionnaire concerning their habits (smoking, brushing type, etc), family background (ethnic group, family dental history) and any previous dental treatments (periodontal, orthodontic, etc). Then the examiner filled in a periodontal examination chart containing the following items:

- The extent and amount of dental plaque accumulation on the buccal, lingual and proximal surfaces of all teeth (4 values per tooth) were assessed visually after the use of a plaque disclosing agent (erythrosine 2%, Dentoplaque Inava [Pierre Fabre Oral Care; Castres, France]) and recorded using the O’Leary Plaque Index (PI): - absence of plaque, + presence of plaque.
- Periodontal Probing Depth (PPD) was measured with a constant-force, computer-assisted periodontal probe accurate to the nearest 0.2 mm. (Florida Probe System; Gainesville, FL, USA). Six sites per tooth were measured (mesio-buccal, buccal, disto-buccal, disto-lingual, lingual, mesio-lingual) on all teeth except third molars. The instrument was kept parallel to the long axis of the tooth for the mid-facial and mid-lingual sites, and as close as possible to the interproximal area for the facial and lingual interproximal sites. PPD was measured from the top of the gingival margin to the point where tissue resisted probe penetration. The Florida Probe enables accurate electronic measurement, digital readout, constant force, full sterilisation of parts in and near the mouth and computer storage of the data. Electronic recording of the data eliminates errors that occur when probe tip markings are read visually and the data are called to an assistant.^18^
- BoP^12^ was recorded at the same time as the PPD measurement, assessing six sites per tooth. A site was considered positive when bleeding occurred 10-15 s after probe insertion. The percentage of BoP positive units was calculated using the formula: (quantity of bleeding sites/quantity of probed sites) x 100.

## Statistical Analysis

Data were analysed using JMP 11.1.1 (SAS Institute for Windows; Cary, NC, USA). Clinical data are from a single sample, collected twice (V1: 2009; V2: 2013). The variation of all periodontal variables, collected at V1 and V2, was analysed by calculating the difference V2-V1 for each subject. McNemar’s test was used to compare the qualitative data; to test statistical significance, Student’s t-test was applied with an α error of 5%.

## RESULTS

### Population Characteristics

The characteristics of this population are summarised in Table 1. All enrolled students completed both visits (n = 25 at V1 and n = 25 at V2).

**Table 1:** Characteristics of the included population at V1 (SD: standard deviation)

		Total (n = 25)
**Age (years)**	Mean ± SD	20.4 ±1
	Median	20
	Min-Max	[19-23]
**Gender**		
Female:male ratio	n:n (%/%)	16:9 (64%:36%)
**Smoking**		
Smoker:former:non smoker ratio	n:n:n (%:%:%)	3:2:20 (12%:8%:80%)


### Oral Hygiene Habits

Table 2 describes oral habits at both visits. In 2009, 92% of included individuals used a manual toothbrush, mostly with medium-type bristles. At V2, 76% of the population had switched to electric brushing, and 100% of the manual toothbrush users used soft-type bristles, which represents a statistically significant change in oral habits (p = 0.0002). Dental floss users increased from 6% to 44%, and mouthwash users represented 20% at both visits. The frequency of toothbrushing remained stable at both visits.

**Table 2:** Oral habits of the included participants at V1 and V2

		V1	V2
**Number of subjects**		25	25
**Brushing frequency/day**	Mean ± SD	2.2 ± 0.5	2.2 ± 0.6
	Median	2	2
	Min-Max	[1-3]	[1-4]
**Brushing duration (min)**	Mean ± SD	2.5 ± 1	2.6 ± 1.3
	Median	2	2
	Min-Max	[1-5]	[1-8]
**Toothbrush type**			
Manual:powered ratio	n:n (%:%)	23:2 (92%:8%)	6:19 (24%:76%)
**Bristle type**			
Soft:medium:hard ratio	n:n:n (%:%:%)	9:14:2 (36%:56:8%)	25:0:0 (100%:0%:0%)
**Flossing**	n (%)	6 (24%)	11 (44%)
**Mouthwash use**	n (%)	5 (20%)	5 (20%)


### Plaque Index

**Fig 1:**
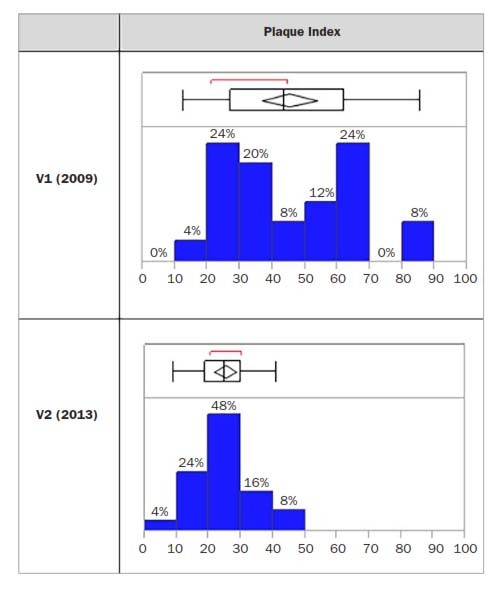
Values and distribution of the plaque index between V1 and V2.

PI values were 45.2 ± 20.3 and 25.1 ± 8.1 for V1 and V2, respectively. At the baseline examination, 96% of individuals exhibited a PI >20. The distribution is as follows (Fig 1):

- 24% of the population exhibited a PI between 20 and 30;
- 20% of the population exhibited a PI between 30 and 40;
- 24% of the population exhibited a PI between 60 and 70;
- 8% of the population exhibited a PI between 80 and 90.

During the second visit, 72% of all individuals exhibited a PI >20, with 48% <30. During the four-year interval, a equalisation of the distribution can be noted with a shift towards lower values than at the first examination.

The mean statistically significant decrease during the four-year period was -20.10% (p < 0.0001) (Table 3).

**Table 3:** Percent change of the plaque index and the bleeding on probing from V1 to V2 (ΔPI, ΔBoP)

	ΔPI	ΔBoP
Sample	25	25
Mean	-20.10	-7.92
Standard deviation	17.82	7.89
95% CI upper limit	-12.74	-4.66
95% CI lower limit	-27.45	-11.18
Median	-16.97	-6
Minimum	-57.15	-27
Maximum	21.42	5
p-value	<0.0001*	0.0004*


### Bleeding on Probing 

**Fig 2:**
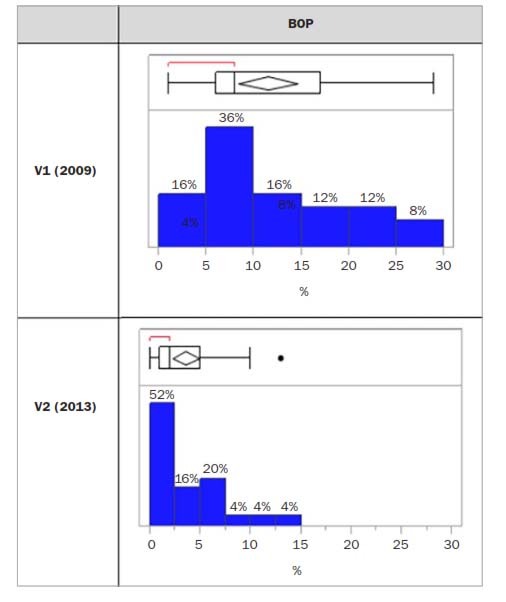
Description of the BoP values at V1 and V2.

Mean BoP decreased from 11.5% ± 7.6 to 3.6% ± 3.2 between the two visits (Fig 2). The reduction in BoP of -7.92% was found to be statistically significant (p = 0.0004) (Table 3). 

In 2009, the BoP ranged from 0 to 30% (Fig 2), but most individuals (68%) presented a BoP <15%, with the largest group (36%) showing from 5-10%. The mean BoP value was 11.5 ± 7.6. 

In 2013, BoP ranged from 0% to 15%, with a majority of individuals (52%) showing a BoP score from 0 to 3%. 

### Periodontal Probing 

At V1, the mean PPD for all probed sites (4133 sites at both V1 and V2) was 1.487 ± 0.55 mm. At V2, it was 1.438 mm ± 0.460 mm (Table 4), which was a statistically significant decrease (ΔPPD global) of -0.049 mm (p < 0.0001) (Table 5). As for gingival recession (GR), the mean scores for V1 and V2 were 0.0038 ± 0.08 mm and 0.0054 ± 0.06 mm. The difference was not statistically significant (p > 0.1), and the values were very close to 0. Thus, GR was not considered a relevant indicator in this study.

**Table 4:** Periodontal pocket depth in mm at V1 and V2, as recorded by an electronic probe

		PPD V1	PPD V2
No. of observations	n	4133	4133
	Mean ± SD	1.487 ± 0.55	1.438 ± 0.46
	Median	1.4	1.4
	Min-Max	[0.4-3.8]	[0-3.2]


**Table 5:** Description of the global change in periodontal pocket depth (mean score of 4133 probed sites)

	ΔPPD global
Sample	4133
Mean	-0.049
Standard deviation	0.594
95% CI upper limit	-0.031
95% CI lower limit	-0.068
Median	0
Minimum	-2.6
Maximum	2.2
p-value	<0.0001*


## DISCUSSION

In this study, the impact of reinforced OH on a population of healthy dental students was investigated using the three most common clinical parameters: the plaque index, bleeding on probing and PPD.

The sample size (25 students) may seem small, but we used the total probing sites in order to increase the statistical power of analysis.

In this study, the mean sulcus depth at V1 was in accordance with previously published data by Vacek et al,^18^ who found a mean sulcus depth of 1.14 ± 0.49 mm. After four years of dental curriculum, the present sample showed a slight but statistically significant global decrease of sulcus depth (0.049 mm decrease, p < 0.0001). The latter was not associated with an increase of GR, as this difference at V1 and V2 was not significant. Furthermore, the mean scores of GR were too low to be considered for interpretation. The main changes in OH habits were the widespread adoption of a power-driven toothbrush and, to a lesser extent, the use of an interdental cleaning aid. These two elements could have contributed to the observed decrease. The statistically significant decrease of sulcus depth, although slight, can be beneficial to periodontal health, as a shallower sulcus presents less risk for the development of periodontopathogenic biofilm. 

Furthermore, the detection of this slight improvement in periodontal status was possible with the use of an electronic probe, as it enabled the examiners to obtain more accurate measurements, which could not be achieved by using a manual probe (accurate to the nearest 0.5 mm). When Gibbs et al^6^ evaluated the Florida Probe, the average standard deviation of repeated pocket depth measurements between three different examiners for the Florida Probe with constant force and accurate electronic measurement was 0.58 mm. This represents a 0.24 mm improvement over the common manual probe. These results were also confirmed by Deepa et al,^4^ who compared the Florida Probe and a conventional UNC-15 probe.^4^

Concerning PI and BoP, this study clearly demonstrated a statistically significant improvement for both parameters (20% decrease of PI, p<0.0001; 79% decrease of BoP, p = 0.0004), which can be explained by the OH awareness of the students acquired during their undergraduate studies. These results are similar to those found by Wagle et al,^21^ who compared oral health status in a population of dentists to that of a population of laypersons. The study concluded that dentists presented a better periodontal status and better self-reported oral health behaviours than the laypersons.^21^ However, the mean recorded PI >20% at V2 could still be considered elevated, considering the high level of OH performance of the sample. Thus, despite a change from 6% to 44%, interdental cleaning was still lacking in more than half of the sample. Without proper interdental cleaning, it is difficult to decrease PI values. 

Moreover, in the present study, the percentages of individuals showing at least one site with PPD >3 mm decreased from 44% at V1 to 8% at V2 (data not shown). This shows that the overall improvement in periodontal status diminished the need for periodontal treatment in 36% of the study population.

The choice of our population, mostly young Caucasians with high periodontal awareness, might be a bias criterion when trying to extend the results to the general population. However, the fact that this specific population was exempt from any periodontal disease also reduces the intra- and inter-examiner discrepancies.^4^,^11^

## CONCLUSION

This study showed a statistically significant improvement of the mean PPD (0.05 mm) after four years of dental studies and reinforced OH methods. However, the clinical impact of this slight gain in periodontal health could be debated. Further studies should be conducted to determine whether this decrease in sulcular depth can reduce the risk of developing periodontal diseases.
